# P-1501. Intracellular Activity of Delafloxacin Against Small Colony Variant *Staphylococcus aureus*

**DOI:** 10.1093/ofid/ofae631.1670

**Published:** 2025-01-29

**Authors:** Kristen L Tudahl, Hailey Thompson, Megan Wimmer, Brian Buss, Jeannina Smith, Adriana Rosato, Cecilia Volk

**Affiliations:** University of Wisconsin Madison School of Pharmacy, McFarland, Wisconsin; Mayo Clinic, Rochester, Minnesota; UW Health, Madison, Wisconsin; UW Health, Madison, Wisconsin; University of Wisconsin Department of Medicine Division of Infectious Diseases, Madison, Wisconsin; MaineHealth Institute for Research, Scarborough, Maine; University of Wisconsin-Madison, Madison, WI

## Abstract

**Background:**

Small colony variants (SCVs) of *Staphylococcus aureus* (SA) have significant intracellular persistence. Fluoroquinolones demonstrate intracellular activity and are often utilized to treat SCVs. Delafloxacin is a fluroquinolone with increased potency at a low pH, which could lead to enhanced intracellular activity. This study evaluates delafloxacin activity against intracellular MSSA and MSRA SCV isolates.

**Figure 1.**
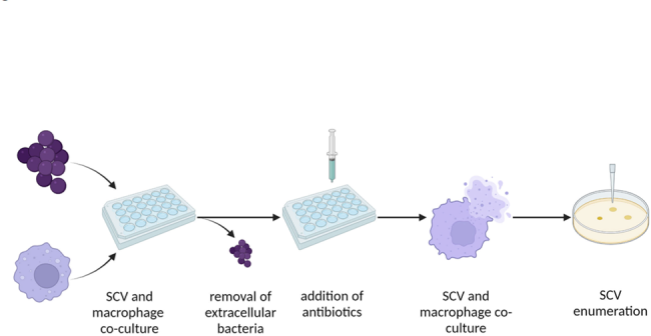
Illustration of SCV co-culture methods

**Methods:**

Six clinical SCV-SA isolates were co-cultured with human macrophages for 1 hour, allowing for phagocytosis (Fig 1). The remaining extracellular bacteria were removed and antibiotic treatments of delafloxacin, levofloxacin, gentamicin, cefazolin, or vancomycin were added. After 5-hour and 24-hour incubation periods, supernatants were collected for extracellular colony forming unit (CFU) enumeration. Macrophages were washed, lysed, collected, and plated for intracellular bacterial CFU enumeration. All conditions were repeated in triplicate and compared using a Mann Whitney U test.

**Figure 2.**
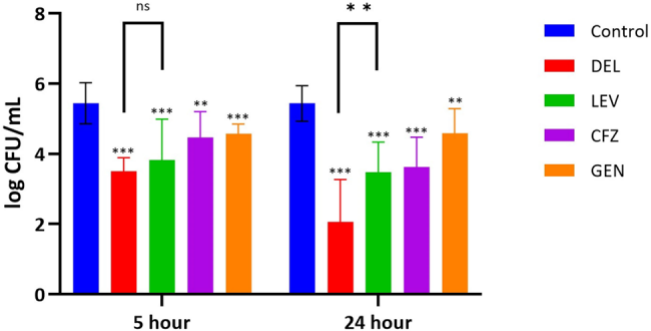
Intracellular SCV-MSSA CFUs

**Results:**

Delafloxacin resulted in a 1.9-log reduction in intracellular CFUs (p< 0.001) at 5 hours and a 3.4 log reduction (p< 0.001) at 24 hours compared to antibiotic-free control for MSSA strains (Fig 2). Similarly, levofloxacin resulted in a 1.6-log (p< 0.001) and 1.9-log (p< 0.001) reduction in CFUs after 5 and 24 hours, respectively. Delafloxacin was more effective at clearing intracellular bacteria than levofloxacin at 24 hours (p= 0.004). In MRSA-SCV isolates, delafloxacin demonstrated CFU reduction of 2.4-log (p< 0.001) at 5 hours and 4.7-log (p< 0.001) at 24 hours compared to antibiotic-free control (Fig 3). Levofloxacin demonstrated a 1.6-log (p< 0.001) reduction at 5 hours and 1.5-log (p= 0.034) reduction at 24 hours. In comparison to levofloxacin, delafloxacin led to greater reductions in MRSA CFUs at both 5 hours and 24 hours of incubation, 0.85-log (p= 0.028) and 3.2-log (p< 0.001) respectively.

**Figure 3.**
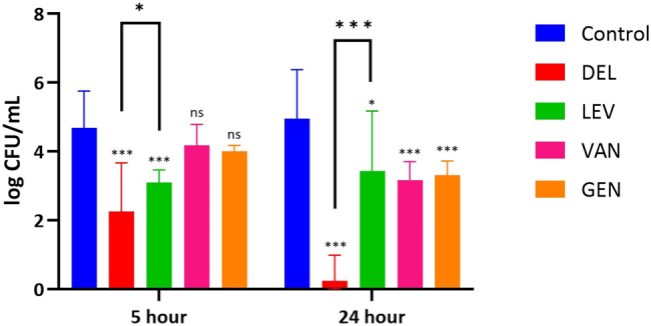
Intracellular SCV-MRSA CFUs

**Conclusion:**

The results demonstrate delafloxacin having greater intracellular activity than levofloxacin and other comparator antibiotics against both MSSA and MRSA SCVs. These results support using delafloxacin to treat recurrent or chronic infections caused by either MSSA or MRSA SCVs and suggest that the observed efficacy may be a result of enhanced intracellular activity.

**Disclosures:**

**All Authors**: No reported disclosures

